# Stressors and coping strategies among single mothers during the COVID-19 pandemic

**DOI:** 10.1371/journal.pone.0282387

**Published:** 2023-03-08

**Authors:** Himawari Wakai, Nobutoshi Nawa, Yui Yamaoka, Takeo Fujiwara

**Affiliations:** Department of Global Health Promotion, Tokyo Medical and Dental University, Tokyo, Japan; Province Health Service Directorate, Technical Support Unit (TSU), NEPAL

## Abstract

**Objectives:**

Increased levels of stress have been reported among parents due to the factors associated with the COVID-19 pandemic. Although social support is known as a protective factor for the stressors, restrictions due to the pandemic could influence the provision and forms of social support. To date, few qualitative studies have examined the stressors and coping strategies in detail. In particular, the role of social support for single mothers during the pandemic remains largely unclear. The purpose of this study is to explore the stressors and coping strategies of single parents during the COVID-19 pandemic, with a focus on social support as a coping strategy.

**Methods:**

In-depth interviews with 20 single mothers were conducted in Japan between October and November 2021. Data were deductively coded using thematic coding based on codes related to stressors and coping strategies, focusing on social support as a coping strategy.

**Results:**

Most interviewees recognized additional stressors after the COVID-19 outbreak. Five stressors were mentioned by the participants: (1) fear of infection, (2) financial concerns, (3) stress caused by interactions with their children, (4) restrictions on childcare facilities, and (5) stress related to staying at home. The major coping strategies were (1) informal social support from family, friends, and coworkers, (2) formal social support from municipalities or nonprofit organizations, and (3) self-coping strategies.

**Conclusion:**

Single mothers in Japan recognized additional stressors after the COVID-19 outbreak. Our results support the importance of both formal and informal social support for single mothers, either in-person or online, to cope with stress during the pandemic.

## 1. Introduction

Even before the COVID-19 pandemic, single mothers were known to be under high stress [1–3]. Thus, the current pandemic may have further exacerbated the vulnerability of single mothers. Several studies conducted during the pandemic have reported that single parents had higher levels of stress than those in two-parent households [4–6]. Recent studies also suggested that potential stressors for single parents during the pandemic included increased responsibilities at home [6, 7], changes in work patterns (shorter or increased working hours) [6], financial hardship, unemployment and decreased face-to-face social support due to social distancing measures [6–10]. In particular, compared to mothers in two-parent households, working single mothers shouldered more responsibilities in terms of childcare, homeschooling, housework, and paid work alone during the pandemic [6], which suggests the need for research focusing on additional stressors of single mothers. Further, studies conducted pre-COVID-19 pandemic reported that single mothers used more coping strategies such as “acceptance of responsibility for problems” and “positive reappraisal of stressful situations” than married mothers [11]. However, the detailed nature of stress and coping strategies of single mothers during the pandemic had not yet been investigated.

Social support is defined as “any process through which social relationships might promote health and wellbeing [12],” and includes (1) emotional, (2) instrumental, (3) informational, and (4) appraisal supportive behaviors [13]. Previous studies have shown that single mothers living in multi-person households (and therefore more likely to receive social support) were less stressed about balancing childcare and work than single mothers living alone with their children [7]. However, social distancing policies during the pandemic have greatly reduced face-to-face social interactions. Thus, the reduction in social interactions due to the pandemic may have affected the coping strategies of single mothers, especially those that involve relying on maternal social support. To date, such coping strategies to counter the stressors of single mothers during the pandemic have also not been explored.

While high rates of children living in single-parent households have been reported in certain Western countries since before the pandemic [14], the unemployment rate for single mothers in the US more than tripled in the early stages of the COVID-19 pandemic [9, 10]. In Japan, single mothers might feel particularly challenged after the pandemic, given the high relative poverty rate (50.8%) among Japanese single parents compared to the average number in OECD countries (31.9%) [15, 16].

In this study, we aimed to explore the stressors and coping strategies of Japanese single mothers during the COVID-19 pandemic, with a particular focus on social support as a coping strategy.

## 2. Methods

### 2.1. Sampling and data collection

Between October and November 2021, in-depth interviews were conducted with 20 Japanese single mothers across Japan with children under the age of 18 years old. Purposive sampling was considered to ensure a variation of cases, such as the regional diversity of the participants. The first 18 participants were recruited through the personal connections of the first author of this study. Following that, we asked the participants to suggest other mothers who might be interested in participating in this study as a snowball sampling. That is, we used snowball sampling to further expand the study participants from people known by our personal connections. The subsequent participants were contacted via social media service or phone call, and two agreed to participate in this research. All participants provided written or digital informed consent. This research was approved by the Research Ethics Committee of Tokyo Medical and Dental University (approval M2021-176-02). More information on data collection is described in S1 Appendix.

### 2.2. Data analysis

We transcribed the interview data recorded in Japanese. To ensure the confidentiality of participants, their names and other identifying information were anonymized. Then, each transcript was coded using the software MAXQDA Plus 2020 (VERBI Software 2019). Data were deductively analyzed using thematic coding based on codes related to: (1) stressors during the COVID-19 pandemic, and (2) the corresponding coping strategies.

Regarding coping strategies, data were coded with a particular focus on social support. They were categorized into sub-groups according to types and sources of social support (i.e., informal and formal social support). These themes were developed based on the literature on social support [12, 13, 17, 18]. Author HW initially led the coding process and after creating the initial codebook for stressors and their coping strategies, authors HW, NN, and YY organized the code lists and finalized the codebook.

## 3. Results

Table 1 shows the characteristics of the 20 participants. All participants were Japanese single mothers with at least one child under the age of 18 years old. More than half reported having two or more children living with them. The geographical distribution of residence of the participants were: Kagoshima, a rural area in the southern part of Japan (45%); Ibaraki, a suburban region close to Tokyo (25%); Tokyo (25%), and Aichi, a city in Central Japan (5%).

**Table 1:** Characteristics of Japanese single mother participants (n = 20).

Variables		Number of participants (n = 20)	%
**Number of children (under the age of 18 years old) living together**	1	9	45
	2	5	25
	3	3	15
	4+	3	15
**Area of residence**	Kagoshima	9	45
	Ibaraki	5	25
	Tokyo	5	25
	Aichi	1	5

While the levels of perceived stress varied among the participants, most recognized additional stressors after the COVID-19 outbreak. The five major stressors for single mothers during the pandemic were restrictions on childcare facilities, stress related to interactions with their children, financial concerns, fear of infection, and stress related to staying at home (Table 2).

**Table 2:** Stressors among single mothers during the COVID-19.

Q3: Stressors during the COVID-19	Stress types	Summary of main stressors	Representative quotes
Restrictions on childcare facilities	Stress related to the places to leave their children	Participants stated that they felt stressed over the issue of where to leave their children during the school closure. Some of them were also concerned about their children staying at home alone while they were out of home.	The afterschool childcare facility asked the upper elementary grades to stay at home alone as possible ("Orusuban"), …Even during the summer holidays, they asked not to use the facility as possible. [SM1]
	Concerns for leaving children alone at home	Participants expressed concern about their children being left alone at home when they go out.	[My work] has been in business without being affected [by the pandemic], so it was not easy for me to take time off. . . I was worried that if my children got injured while staying at home alone, I was ready to go home immediately, but I also had responsibilities for my work. I couldn’t leave any holes in my schedule, so it was a little hard to take time off easily. [SM7]
	Stress related to outings/shopping with children	Participants also reported feeling stressed of bringing their children to shopping or outings due to the restrictions on childcare facilities.	I used to decide to spend one of my two days off a week running my errands, but the preschool asked me to let my child have a day off on weekdays when the mother was off. So, I couldn’t do my errands anymore…During the peak of infection in winter, I was really frustrated with the stress of having to take my child to the bank, city hall, and other crowded places in the middle of the COVID-19 pandemic, and the stress of not being able to get things done because of my child. [SM19] It’s hard to do even a single shopping trip, and people say that I should just leave them at home, but I’m the only one adult in the household. So, I couldn’t leave them at home, and I guess it was pretty hard. [SM9]
Stress related to interactions with their children	Increased frequency of family quarrel/ scolding at children	Participants stated that the disruption of their children’s lifestyles due to school closure was also stressful. Particularly, many participants were stressed about their children’s excessive use of games and smart phones. Participants stated that quarrels with their children and scolding increased due to the COVID crisis. Some respondents felt stressed due to increased fighting among children.	I think the biggest thing was the stress of my daughter due to restrictions on club activities and other things. She took that stress home with her. Sometimes, we ended up saying things to each other that didn’t need to be said. I guess we argued a lot more [compared to before the pandemic]. [SM17] [My daughter] spends longer hours watching TV or on YouTube, and I get angry at her for that. I guess we’re both a bit stressed out about it. [SM1]
Financial concerns	Decreased amount of workload/ Income reduction/ job loss	Regarding work situations, some participants were negatively affected by the pandemic.	In July of last year, I was actually fired, or I would say, being terminated from employment. . . It was not like I just got a job there, but it’d been less than a year, and I was probably the one who was easiest to tap on the shoulder [be pressured into retirement]. But since I was working part-time, they didn’t even cover my insurance. [SM14] When I contacted them for an interview, they were not in a condition to hire someone due to the pandemic. And they asked, "What happens when [your children] have online classes? What will you do?" [I thought that] the older kids are fine, but three of them will definitely need a parent. Finally, I was told, "We are sorry [we cannot hire you]." [SM9]
	Pressure for shouldering economic responsibilities by themselves (Concerns for future income reduction/ not being able to take a day off)	Participants stated that they felt pressure, stress, and anxiety about being a single parent and being responsible for their livelihood. They also mentioned the stress of not being able to take time off work under any circumstances and the anxiety about the impact of COVID-19 on their income.	I felt the pressure of having to work because I was a single parent, and well, it was different from a family with a husband. . .during the COVID-19 crisis, I felt the inconvenience of being a single parent. [SM11] Since I was the only one earning money, I couldn’t take time off, and I didn’t want to bother people by telling them about it. I couldn’t show my weakness to others either, and I started to feel emotionally down from around July. In the end, I could no longer do my job properly, nor could I do my housework. [SM1]
	Increased cost of food/living	Participants expressed their stress and anxiety over the increased cost of food and living expenses due to school closure and self-restraint.	While they [my children] are in school, they don’t have to snack or anything, but when they are at home all day, the amount of food they eat is totally different, so I need to cover for that. . . I need to cover the cost of food, and prepare for the whole day. . . and I am worried about that. That’s the biggest burden. So, it’s always the cost of living. The burden that I bear is. [SM7] I talked to the people in charge of public assistance and told them that we were having a hard time affording food since we were staying at home, but they just told us that the amount of money was fixed and we had to manage within it. [SM3]
Fear of infection	Fear of oneself getting infected	Participants mentioned fear of infection for themselves or family members living with them. Some were worried about their children if they themselves were infected.	I wondered who would take care of my kids if I were to become seriously ill. Similarly, I wondered who will support us when my kids or I get infected. [SM12]
	Fear of family members getting infected	Participants reported stress and anxiety about people’s perceptions of them and their children when they had cold symptoms, and about the public’s reaction in case of infection.	My mother, who lives with me, is on dialysis. So, the most stressful thing for me was the risk of infection in case I bring the virus into the house. [SM16] When the number of cases increased in my hometown, I was worried about when would I get infected and what would I do if my children got infected. [SM10]
Stress related to staying at home	Reduced opportunities for outings and social interactions	Over half of the participants reported stress from staying at home all the time due to reduced opportunities to go out. Some of them felt isolated due to the reduced opportunities to interact with the people around them. In addition.	Everything has changed so differently now, and moreover, there are three people on my shoulders, and I wondered if I get infected, or when will there be such an incident in elementary school. My heart was always pounding. So, because of that, I became less involved with people. I did feel isolated and lonely. [SM12] I can no longer go out to lunch with my friends as I used to do, and school events, nursery school sports events, and other social events are separated into different times. . . It’s almost like, well, I don’t know, I feel a little isolated. [SM3]

### 3.1. Stressors during the COVID-19 pandemic

#### 3.1.1. Restrictions on childcare facilities

Almost half of the single mothers in this study recognized that restrictions on childcare facilities such as nursery schools, schools, or afterschool childcare facilities, were significant stressors during the pandemic. In particular, many working single mothers were concerned about their children while they were not home: “[My work] has been in business without being affected [by the pandemic], so it was not easy for me to take time off… I was worried that if my children got injured while staying at home alone, I was ready to go home immediately, but I also had responsibilities for my work. I couldn’t leave any holes in my schedule, so it was a little hard to take time off easily” [SM7]. Further, some participants had difficulties when it comes to going on outings with their children (Table 2).

#### 3.1.2. Stress related to interactions with their children

Stress related to interactions with their children during school closure also affected the perceived stress of single mothers. For example, the frequency of family quarrels generally increased during the COVID-19 pandemic: “I think the biggest thing was the stress of my daughter due to restrictions on club activities and other things. She took that stress home with her. Sometimes, we ended up saying things to each other that didn’t need to be said. I guess we argued a lot more [compared to before the pandemic]” [SM17].

#### 3.1.3. Financial concerns

Over half of the single mothers reported financial concerns as a stressor during the pandemic. During the pandemic, changes in job environments was a major stressor. These include job loss, reduced working hours, and job hunting. One mother stated: “In July last year, I was actually fired, or I would say, being terminated from employment… It was not like I just got a job there, but it’d been less than a year, and I was probably the one who was easiest to tap on the shoulder [be pressured into retirement]. But since I was working part-time, they didn’t even cover my insurance” [SM14]. Similarly, stress related to job hunting during the pandemic was also reported (Table 2).

Predominantly, working single mothers stated they felt the pressure of shouldering economic responsibilities by themselves (Table 2). Other types of financial stress including the increased cost of food and living expenses during school closure were also reported (Table 2). More discussion on fear of infection and stress related to staying at home are found in S2 and S3 Appendices.

Of note, stressors may differ between single mothers with children under the age of 5 years and those with children over the age of 5 years. For example, most single mothers with children under the age of 5 years reported feeling stressed due to the reduction of outdoor play areas for their children during the pandemic. However, additional research is needed as the sample size is not sufficient for data analysis stratified by age.

### 3.2. Coping strategies

#### 3.2.1. Support and connection from family members

A majority of the single mothers in this study received family social support from their parents or siblings during the pandemic. Extended family members extended support by temporarily taking care of the children, providing advice in times of trouble, and interacting with them regularly either in person or online. More results on family support are found in S4 Appendix.

#### 3.2.2. Support and connection from friends and acquaintances

The participants perceived friends and acquaintances as primary sources of support during the pandemic. Some single mothers pointed out the importance of mom friends–friends who are mothers themselves and who understand when you talk about parenting–during school closure: “During periods of self-restraint or school closures, we would take care of each other’s children with my single mom friend, by shifting our workdays a little. In my case, I had to work on a reduced schedule [due to the COVID-19 restrictions], so I only went to work once or twice a week. So, I took care of her children on my days off and vice versa” [SM14].

Coworkers having children of the same age also appeared to play a key role in relieving the stress of working single mothers: “The best thing was to go to the workplace and talk to the mothers who work there. It was best to talk to people [in the workplace], rather than friends because this job is the foundation for my life. Asking the people at work, "So how’s it going on your end?" That’s when I felt like, "Everyone is trying hard, so I should try my best too!"” [SM12].

#### 3.2.3. Support and connection from nonprofit organizations (NPOs)

Some single mothers, especially those who did not receive support from their family members, reported receiving support from NPOs. Specifically, the use of meal provisions, children’s cafeterias, and free tutoring were frequently mentioned. One single mother said: “We received some food from a non-profit organization, less than enough to cover us for a day…but the fact that we received something was a big help, and it really made me feel better, so that helped me out a little…Since the COVID-19, we don’t get together and have a meal at the children’s cafeteria like we used to. But every month or two, they invited one family to come and eat curry and rice…I felt quite reassured by them. Relatives… like dependable relatives” [SM3]. Such connections with NPOs during the pandemic appeared to ease the sense of isolation in some cases (Table 3).

**Table 3:** Coping strategies among single mothers during the COVID-19 pandemic.

Coping strategies	Summary of main coping strategies for the stressor	Representative quotes
Support and connection from family members	Participants mentioned that they felt relieved when asking their parents to keep their children at parents’ home.	It’s almost like our parents are raising my child. I owe a lot to my parents. I can’t go to work without them. [SM19] My kid’s grandma and grandpa take care of her once a week, preparing meals for her. . .They bought me something to eat, or sometimes they cooked a meal and brought it to us. Well, I think it was very helpful to have meals prepared for us while trying not to be in contact for a long time. [SM11] She [my daughter] goes [to my parents’ home] on Fridays after school, and I pick her up on Saturdays when I finish work, and she comes home. I feel that we can let off steam in this way. [SM17] When we were on a tight budget, we went to my parents’ home to eat. [SM14] When I felt like suffocating at home, I sometimes asked my mother to watch my kids for a while. . . . Sometimes after the COVID-19, when I felt I was reaching my limit, the best thing for me was being able to ask my mother for help. [SM12]
Support from friends and acquaintances	Participants mentioned that they were helped by their mom friends to watch their children.	During periods of self-restraint or school closures, we would take care of each other’s children with my single mom friend, by shifting our work days a little. In my case, I had to work on a reduced schedule [due to the COVID-19 restrictions], so I only went to work once or twice a week. So, I took care of her children on my days off, and vice versa. [SM14] Instead of people close to me, I talk with mothers who may have the same problems. They don’t ask too deep questions, do they? They’re just colleagues. They don’t ask me things like, " How’s your family situation?" So, it’s very easy to talk to them. It’s great to have mothers with children of the same age who keep a good distance. [SM19] Even with friends that I used to only talk to on the phone, I can talk to them face to face on video phone or through a screen. That alone made me feel like I could process my feelings better, and I think that was something I was glad about. [SM4] After the kids go to bed, on a Saturday night around 11:00 p.m., we decide on a time of one hour. We’d have a group call on the LINE with about four people, and those who could drink would bring drinks, and those who couldn’t would bring soft drinks, and we’d talk about what had happened recently, what happened during the school closure, what happened with the lessons, and so on. I felt that we both reaffirmed the importance of the network connection when we talked to each other. I think it’s important. [SM13] The best thing was to go to the workplace and talk to the mothers who work there. It was best to talk to people [in the workplace], rather than friends, because this job is the foundation for my life. Asking the people at work, "So how’s it going on your end?" That’ s when I felt like, "Everyone is trying hard, so I should try my best too!" [SM12]
Support and connection from NPOs	Participants reported that support from NPOs had eased their concerns about their living conditions. Specifically, they were using support such as the provision of food, children’s cafeterias, and free tutoring.	We received some food from a non-profit organization, less than enough to cover us for a day. . .but the fact that we received something was a big help, and it really made me feel better, so that helped me out a little. . . Since the COVID-19, we don’t get together and have a meal at the children’s cafeteria like we used to. But every month or two, they invited one family to come and eat curry and rice. . . I felt quite reassured by them. Relatives. . . like dependable relatives. [SM3] We receive food once every two months. . .It’s only my income. But since I don’t work as a full-time employee, I don’t receive a sizeable amount of salary. So, it’s very helpful. [SM17] There are people who support single-mother families, right? These people come to see us occasionally. Although the number of visits has decreased a little, it has been very helpful. . . . Since I live far from my parents’ home, I don’t rely on them at all, so I’m completely on my own. So, it’s a bit of a relief. [SM7] Instead of feeling isolated, I’ve been able to feel reassured through my connection with the children’s cafeteria. [SM3] They [NPO staff] tell me what they’ve experienced [on parenting]. And if they don’t have the experience, they still catch up with me by researching and studying [regarding the issue I was facing]. . .You know, I used to think that I couldn’t tell anyone about my problems, and that I was always retreating into my shell, but since the pandemic, I’ve found a place where it’s okay to talk about these things. I had known about the organization and its activities, but it was really only recently that I stepped into the place. [SM9] Honestly, it’s the same even after the pandemic, but whenever I check TV, radio, or social media, I find that people in the Tokyo metropolitan area are running children’s cafeterias or food drives. But I think there are still many rural areas where such activities are still lacking… I think most of the food drives for single mothers and single fathers are held in the cities. That’s why I often feel that they don’t reach the people who really need it, and that the necessary support environment is not in place for those who need it. [SM13]
Support from municipalities	Participants stated that their stress was reduced by utilizing various public systems and supports, including maternity allowance and benefits from the local government. On the other hand, several respondents also mentioned that although they were eager to use the service, they were not utilizing the service well because of lack of information about the support and lengthy application process.	I appreciate the government [support], though. Regardless of the COVID-19 pandemic, there is maternity allowance available, and I am grateful for that. I am now working part-time for short hours because of that. [SM5] It’s also that I don’t know if there are any [supports available]. Maybe I could have used it [public support] more if there had been anything available, but I don’t even know the type of support available to me…I don’t know if there are any opportunities to learn about such things, or places to consult. So, I’m hoping there’s a place that could tell me [about those services that I can utilize]. [SM17] I think it costs more for families with older children…For the little kids, there seems to be a lot of temporary childcare, sitters, and many supports like that, but when they get older, I don’t think there’s anything for them anymore. [SM17] The money came in at a time when I was feeling like I’m in trouble. So, I was very relieved! These days, I’ve been draining my savings, or rather, my very small savings, but I can’t afford to drain it. I had been thinking, "What’s really going to happen?" But anyway, it helped me a lot. [SM12]
Limit in-person interactions to family members and coworkers only	Some participants limited their in-person interactions to only those they interact with on a daily basis, such as family members or coworkers.	During the period of self-restraint, I sometimes asked my family to buy things for me. . . I can ask people [i.e., family members] I have had contact with before the pandemic. . . I thought it would be a bit risky to make a new contact and ask for help. [SM4]
Self-coping strategies	Doing enjoyable activities at home (redecorating, cooking, using food delivery services, watching streaming services, and drinking)	I’m going to cook dinner with my son, and make something different, like cooking something on a hot plate. . . Doing things with my son that we could do while playing. [SM4] Even if I talk to someone, it won’t make Coronavirus go away. It’s not going to change the current situation. So, what do I do now? It was better for me to drink and feel tipsy. [SM12]
	Walking/Exercise	I like to exercise. I’m playing basketball right now… It’ s like I’m getting rid of stress by exercising. [SM20] My daughter and I were feeling depressed in the house, so we decided to go for a walk. So, I cycled with my daughter to a park in our neighborhood, which is a bit far from our house. Walking. I thought it felt good to exercise… But that’s all I could do now. Honestly, if you ask me if I’ve been able to release my stress, I’m not sure. [SM19]

Further, NPOs could also provide emotional support for single mothers by providing advice and information (Table 3). Meanwhile, limited access to NPO services was also reported, especially among single mothers in rural areas (Table 3).

#### 3.2.4. Support from municipalities

Over half of the participants responded that their stress was reduced by seeking help from various public systems, including receiving maternity allowance and benefits from the local government. While most single mothers referred to the COVID-19 special benefit payment, a few commented on public services such as maternity allowance, which had been available from even before the pandemic (Table 3). Although some single mothers actively sought public support, several respondents perceived barriers to using the services. For example, despite being eager to use the service, some single mothers were not utilizing the services due to a lack of information about the support, coupled with a complex and lengthy application process (Table 3). Further, some single mothers perceived that available social support decreases with age, despite the higher cost of raising older children compared to younger children (Table 3). Other results on limiting in-person interactions and self-coping strategies are included in S5 and S6 Appendices.

## 4. Discussion

Our findings suggest that Japanese single mothers experienced additional stressors during the COVID-19 pandemic. The five major ones are restrictions on childcare facilities, stress related to interactions with their children, financial concerns, fear of infection, and stress related to staying at home.

Stress related to restrictions on childcare facilities was frequently reported by the participants in this study. The results are consistent with those from a recent study in Japan which showed an increase in parenting stress due to school closures during the pandemic [19]. Particularly, employed single mothers struggled with juggling work and childcare during school closure. Notably, work-life conflict among employed single mothers, such as the lack of flexibility at work to meet family needs, has been identified as a stressor even before the pandemic [20, 21]. Restrictions on childcare facilities seemed to have increased childcare burden of single mothers, creating more challenges in balancing work and childcare.

Stress related to school closures and stress caused by interactions with their children during the pandemic appeared to have increased among single mothers. Especially during school closures, disrupted daily routines among children, especially increased screen time, seemed to trigger family quarrels. Similarly, previous research has also identified behavioral issues among children during school closures due to the COVID-19 pandemic [22, 23]. Therefore, more support for single mothers is needed, including ensuring temporary childcare places during school closures or the expansion of online counseling systems for mothers.

Consistent with recent research findings [6, 8–10], financial concerns were reported as stressors among the single mothers. For example, single mothers reported stress caused by decreased working hours or income, job loss, and difficulties in finding a new job during the COVID-19 pandemic. The results were also in line with the trend in Japan when it comes to the employment situation, where women were hit harder by the pandemic than men [24]. According to a survey before the pandemic, 50.8% of single-parent households in Japan lived in relative poverty [15], and 43.8% of employed single mothers were non-regular workers [25]. Thus, single mothers, who were initially at higher risk of unemployment, seemed to have been more affected by the pandemic. Therefore, continuous financial support for single mothers is necessary, especially for those who were financially hit by the pandemic. More discussion on fear of infection is found in S7 Appendix.

The main coping strategies among Japanese single mothers during the pandemic were: (1) informal social support from family, friends, and coworkers with children of the same age, (2) formal social support from municipalities and NPOs, and (3) self-coping strategies.

Friends and coworkers with children of the same age are essential sources of social support for single mothers. A nationwide survey in Japan in 2016 revealed that 33.3% of single mothers reported acquaintances and neighbors as the most common sources of advice [25]. Likewise, friends, especially those who have children of the same age, were a primary source of social support among single mothers during the pandemic. For example, some mothers reported asking friends for emergency childcare support when childcare facilities were limited, which allowed them to go to work. Working single mothers tend to share their concerns with their colleagues, especially those with children of the same age, rather than with friends outside of work. Further discussion on informal social support is found in S8 Appendix.

We also found that instrumental support from NPOs may be a protective factor for stress among single mothers during the pandemic. For example, single mothers who received NPO support fell emotionally connected with the NPOs during the pandemic. In the case of single mothers without parental support, NPOs could serve as “relatives” who they can rely on when they need help.

Nonetheless, the barriers hindering access to NPO support were also pointed out. One of the barriers is limited access to NPO support for single mothers in rural areas. Some single mothers in rural areas indicated a lack of access to NPO services in their neighborhoods, unlike in urban cities. Psychological barriers were also reported, including concern about the stigma associated with using NPO services. A previous study reported that single mothers in Japan tend not to actively seek support for fear that their children might be bullied due to discrimination or harmful stereotypes [26]. This might be the case especially where support is only available at specific locations, such as children’s cafeterias. Thus, outreach support that allows single mothers to use the service discreetly may be needed.

The use of support from municipalities also appears to be a key coping strategy. Frequently utilized services include special benefits and employment support during the COVID-19 pandemic. While these instrumental forms of support may have a role in reducing the financial concerns of single mothers, our findings suggest a need for continuous financial support during the pandemic. Further, the barriers to accessing public support were identified: (1) complex application procedures, and (2) a lack of information on services available to single mothers. Horiuchi et al. [27] also pointed out reduced access to public support during the COVID-19 pandemic due to the cancellation of in-person services (health examination for infants/parenting classes) and the avoidance of service users from going out for fear of infection. Active information provision on public support is necessary to increase awareness of the service. Furthermore, a lack of continued public support for single parents has been pointed out by the authors [28]. Therefore, a comprehensive consultation service for single mothers needs to be established to provide seamless support. More discussion on self-coping strategies can be found in S9 Appendix.

This study has several limitations. Given that this is a qualitative study with a limited sample size, findings may not fully represent the stressors and coping strategies for all Japanese single mothers during the pandemic. Also, findings from this study do not reflect the stressors and coping strategies among single fathers since all participants are single mothers. As single fathers may also experience the challenges of juggling both work and childcare during the COVID-19 pandemic, future research in this regard is needed. Another limitation is that the results of this study are based on self-reported experiences and not on a stress scale. Further research using a scale is needed to examine stress changes before and after the pandemic. Finally, interviews with key informants, who serve as sources of social support for single mothers, would have provided a more comprehensive understanding of the current situation.

Nevertheless, this study has several policy implications. First, active information provision on public support through social media and strategic information delivery through collaboration with related NPOs are needed to promote support utilization among single mothers. Also, a comprehensive consultation service needs to be established to enable single mothers to receive appropriate support when needed. This simplification of the public support process will help promote the use of support. The provision of online consultation services may help reduce parenting stress, particularly in the current pandemic climate. For example, in Nerima City, Tokyo, online childcare counseling by nursery staff and nurses was launched after the COVID-19 outbreak [29]. These online services should be expanded to serve single mothers for easy and safe access to services. For single mothers with limited access to the internet, additional support might be necessary, such as a tablet device rental service. For example, in South Korea, a lack of internet access during a pandemic was a barrier for single mothers to receive public support [30]. Finally, continuous financial support for single mothers during the pandemic is important to mitigate the economic impact of the pandemic on this group. Temporary monthly benefits to single mothers, for instance, could reduce the financial pressure among single mothers at financial risk during the pandemic.

## 5. Conclusions

Japanese single mothers recognized additional stressors after the COVID-19 outbreak. Our study highlights the importance of formal support from municipalities and NPOs as well as informal support from family and friends to help single mothers cope with stress during the pandemic. Our findings also suggest the importance of social support both in person and online.

## Supporting information

S1 Appendix Additional information on data collection. (DOCX)

S2 Appendix 3.1.4 Fear of infection. (DOCX)

S3 Appendix 3.1.5 Stress related to staying at home. (DOCX)

S4 Appendix 3.2.1 Support and connection from family members. (DOCX)

S5 Appendix 3.2.5 Limit in-person interactions to family members and coworkers only. (DOCX)

S6 Appendix 3.2.6 Self-coping strategies. (DOCX)

S7 Appendix Additional discussion on fear of infection. (DOCX)

S8 Appendix Additional discussion on informal social support from family members. (DOCX)

S9 Appendix Additional discussion on self-coping strategies. (DOCX)

S10 Appendix Interview guide. (DOCX)
