# Supplementary material for: Stressors and coping strategies among single mothers during the COVID-19 pandemic
Source: PLoS One. 2023 Mar 8;18(3):e0282387. doi: 10.1371/journal.pone.0282387 (PMC9994735; doi:10.1371/journal.pone.0282387)
Supplement: S1 Appendix — (DOCX) [file pone.0282387.s001.docx]

**S1 Appendix. Additional information on data collection**

After obtaining informed consent, all interviews were conducted via Zoom or phone call, except for one participant who chose an in-person interview. Interviews mainly took place via Zoom. However, for participants who had problems with Zoom interviews (i.e., no internet), we conducted interviews by phone with consideration. With permission, all interviews were audio recorded. Each interview was conducted using a semi-structured interview guide and took between 20 to 90 minutes (S10 Appendix . Interview guide). The interview guide included questions on stressful experiences during the COVID-19 pandemic, and their coping strategies with a particular focus on social support. When the answer of the participant was unclear, we checked our understanding to prevent any misinterpretation. Follow-up interview was conducted with one participant because of time constraints in the first interview. Interviews were conducted until data saturation was reached.
