# Supplementary material for: Stressors and coping strategies among single mothers during the COVID-19 pandemic
Source: PLoS One. 2023 Mar 8;18(3):e0282387. doi: 10.1371/journal.pone.0282387 (PMC9994735; doi:10.1371/journal.pone.0282387)
Supplement: S2 Appendix — (DOCX) [file pone.0282387.s002.docx]

**S2 Appendix. 3.1.4. Fear of infection**

Fear of being infected, for either themselves or their family members, was identified as a stressor for some participants. One explained: “I wondered who would take care of my kids if I were to become seriously ill. Similarly, I wondered who will support us when my kids or I get infected?” [SM12]. A mother living with high-risk family members said: “My mother, who lives with me, is on dialysis. So, the most stressful thing for me was the risk of infection in case I bring the virus in the house” [SM16].
