# Supplementary material for: Stressors and coping strategies among single mothers during the COVID-19 pandemic
Source: PLoS One. 2023 Mar 8;18(3):e0282387. doi: 10.1371/journal.pone.0282387 (PMC9994735; doi:10.1371/journal.pone.0282387)
Supplement: S3 Appendix — (DOCX) [file pone.0282387.s003.docx]

**S3 Appendix. 3.1.5. Stress related to staying at home**

The most frequently reported stressor for single mothers during the pandemic was stress related to staying at home. Reduced opportunities for social interactions were major sources of stress, especially for mothers living alone with their children: “Everything has changed so differently now, and moreover, there are three people on my shoulders, and I wondered if I get infected, or when will there be such an incident in elementary school. My heart was always pounding. So, because of that, I became less involved with people. I did feel isolated and lonely” [SM12].
