# Supplementary material for: Stressors and coping strategies among single mothers during the COVID-19 pandemic
Source: PLoS One. 2023 Mar 8;18(3):e0282387. doi: 10.1371/journal.pone.0282387 (PMC9994735; doi:10.1371/journal.pone.0282387)
Supplement: S4 Appendix — (DOCX) [file pone.0282387.s004.docx]

**S4 Appendix. 3.2.1. Support and connection from family members**

For example, a working single mother who lives with her parents asked them to take care of her daughter when the use of childcare facilities was restricted: “It's almost like our parents are raising my child. I owe a lot to my parents. I can't go to work without them” [SM19]. Also, when experiencing stress related to interactions with their children, some single mothers relied on their parents so that they and their children could get some space (Table 3). When it comes to financial concerns, consulting with and seeking help from family members was the most common strategy: “When we were on a tight budget, we went to my parents' home to eat” [SM14]
