# Supplementary material for: Stressors and coping strategies among single mothers during the COVID-19 pandemic
Source: PLoS One. 2023 Mar 8;18(3):e0282387. doi: 10.1371/journal.pone.0282387 (PMC9994735; doi:10.1371/journal.pone.0282387)
Supplement: S5 Appendix — (DOCX) [file pone.0282387.s005.docx]

**S5 Appendix. 3.2.5. Limit in-person interactions to family members and coworkers only**

Some single mothers reported limiting their in-person social interactions to only those with whom they have daily interactions, such as family members or colleagues, in order to cope with their fear of infection. One mother stated: “During the period of self-restraint, I sometimes asked my family to buy things for me... I can ask people [family members] I have had contact with before the pandemic... I thought it would be a bit risky to make a new contact and ask for help” [SM4].
