# Supplementary material for: Stressors and coping strategies among single mothers during the COVID-19 pandemic
Source: PLoS One. 2023 Mar 8;18(3):e0282387. doi: 10.1371/journal.pone.0282387 (PMC9994735; doi:10.1371/journal.pone.0282387)
Supplement: S6 Appendix — (DOCX) [file pone.0282387.s006.docx]

**S6 Appendix. 3.2.6. Self-coping strategies**

The use of self-coping strategies for stress related to staying at home was also frequently mentioned. For example, doing enjoyable activities at home were common coping strategies: redecorating, cooking, using food delivery services, and watching streaming services. Especially, using food delivery appeared to increase specifically during staying at home, but these increased food costs were linked to stress for some single mothers. Besides activities at home, walking or exercise was also reported as a coping strategy for the stress of staying at home: “I like to exercise. I'm playing basketball right now…It’s like I'm getting rid of stress by exercising” [SM20]. However, most single mothers also recognized that these self-coping strategies did not relieve all the stress caused by staying at home during the pandemic. One mother commented: “My daughter and I were feeling depressed in the house, so we decided to go for a walk… I thought it felt good to exercise… But that's all I could do now. Honestly, if you ask me if I've been able to release my stress, I'm not sure” [SM19].
