# Supplementary material for: Stressors and coping strategies among single mothers during the COVID-19 pandemic
Source: PLoS One. 2023 Mar 8;18(3):e0282387. doi: 10.1371/journal.pone.0282387 (PMC9994735; doi:10.1371/journal.pone.0282387)
Supplement: S7 Appendix — (DOCX) [file pone.0282387.s007.docx]

**S7 Appendix. Additional discussion on fear of infection**

Amid the COVID-19 pandemic, women reported higher fear of infection than men^1^. For single mothers, concern about who will take care of their children in case she herself is infected seems to be another source of stress. Fear of infecting their parents was also reported by those living with or frequently interacting with their parents. Consequently, some single mothers in this study restricted their contact only with those whom they have having daily interactions, such as cohabiting family members and coworkers. Therefore, connections with single mothers through outreach and online support might reduce the sense of isolation.

Supplementary reference

^1^Andrade EF, Pereira LJ, Oliveira APLD, et al. Perceived fear of COVID-19 infection according to sex, age and occupational risk using the Brazilian version of the Fear of COVID-19 Scale. *Death studies*, 2020:1-10. doi:10.1080/07481187.2020.1809786
