# Supplementary material for: Stressors and coping strategies among single mothers during the COVID-19 pandemic
Source: PLoS One. 2023 Mar 8;18(3):e0282387. doi: 10.1371/journal.pone.0282387 (PMC9994735; doi:10.1371/journal.pone.0282387)
Supplement: S8 Appendix — (DOCX) [file pone.0282387.s008.docx]

**S8 Appendix. Additional discussion on informal social support from family members**

Family members were the most frequently reported source by single mothers during the pandemic which is consistent with previous findings^1^. In this study, more than half of the participants recognized receiving emotional, instrumental, and informational support from extended family members, including parents and siblings, despite the current social restrictions. Particularly for working single mothers, family social support appeared to have helped them balance work and family responsibilities during the pandemic. A systematic review by Campbell et al.^2^ pointed to the importance of social support from family and friends for single-parent families successfully managing work-life conflict. A study on employed single mothers in the United States also suggests that instrumental social supports, especially from extended family members (i.e., informal childcare support) reduce work-family conflict among employed single mothers^3^.

Supplementary reference

^1^Lumino R, Ragozini G, Vitale MP. Investigating social support patterns of single mothers from a social network perspective. *International Review of Social Research.* 2016; 6(4): 182–194. doi:10.1515/irsr-2016-0021

^2^Campbell M, Thomson H, Fenton C, et al. Lone parents, health, wellbeing and welfare to work: a systematic review of qualitative studies. *BMC Public Health*. 2016;16:188. doi:10.1186/s12889-016-2880-9

^3^ Ciabattari T. Single Mothers, Social Capital, and Work–Family Conflict. *Journal of Family Issues*. 2007;28(1):34-60. doi:10.1177/0192513X06292809
