# Supplementary material for: Stressors and coping strategies among single mothers during the COVID-19 pandemic
Source: PLoS One. 2023 Mar 8;18(3):e0282387. doi: 10.1371/journal.pone.0282387 (PMC9994735; doi:10.1371/journal.pone.0282387)
Supplement: S9 Appendix — (DOCX) [file pone.0282387.s009.docx]

**S9 Appendix. Additional discussion on self-coping strategies**

Regarding self-coping strategies, our study results suggest the perceived importance of moderate physical activities for mental health among single mothers during the COVID-19 pandemic, which is consistent with a recent study on working mothers in the United States^1^. However, most single mothers also perceived that their self-coping strategies do not completely address their own stress. In addition to practicing self-coping strategies individually, improving access to social support is necessary to ease the stress of single mothers during the pandemic.

Supplementary reference

^1^Limbers CA, McCollum C, Greenwood E. Physical activity moderates the association between parenting stress and quality of life in working mothers during the COVID-19 pandemic. Ment Health Phys Act. 2020;19:100358.doi:10.1016/j.mhpa.2020.100358
