# Supplementary material for: Stressors and coping strategies among single mothers during the COVID-19 pandemic
Source: PLoS One. 2023 Mar 8;18(3):e0282387. doi: 10.1371/journal.pone.0282387 (PMC9994735; doi:10.1371/journal.pone.0282387)
Supplement: S10 Appendix — (DOCX) [file pone.0282387.s010.docx]

**S10 Appendix. Interview Guide**

･Thank you very much for taking time out of your busy schedule to talk with me today. I would like to interview you about my research project, “Stressors and coping strategies among single mothers during the COVID-19 pandemic.”

･First of all, I would like to ask you if you have read the materials that I sent to you by e-mail in advance regarding the purpose of this research. Did you find anything unclear?

(If there are any unclear points, explain them)

Now, I would like to briefly explain about this study again. Today, we are planning to have a 60-minute interview. The questions I would like to ask you are about your mental health before and after the COVID-19 and about social support from people close to you. If you have any questions that you prefer not to answer, please feel free to skip them. You can stop the interview at any time, even after the interview has started, and you will not suffer any disadvantage by that.

･This is all about the interview. Do you have any questions? (If there are any questions, answer them.) I would like to record this online interview in order to accurately record the content of the interview, if that is okay with you.

(For face-to-face interview) I would also like to record this interview for the purpose of accurately recording the content of the interview, if that is okay with you.

･Now, let's move on to the interview.

**Pre-interview Questions**

1. Please tell us about your family members who are currently living with you.

**Mental Health before and after the COVID-19**

･First, I would like to ask you about your stress.

1. What do you feel is the most stressful thing during the COVID-19 pandemic?
2. Why do you feel it is the most stressful?
3. When do you think you have felt the most stressed since the COVID-19 pandemic? (i.e., During the school closure period, at present (after the rebound of the infection cases in August 2021)

**Social Support from Close People before and after the COVID-19 pandemic**

･Please tell me about your interactions with the people around you.

1. When you feel worried or stressed at present, please share your experiences, if you have felt better by talking to people around you or having them listen to you.

･What kind of relationship with the person?

･How often and when do you see them?

･How do you keep in touch with them (if online)?

i.e. LINE or ZOOM

1. Compared to before the COVID-19 pandemic, how have your opportunities to talk or consult with people around you changed?

･Do you feel any change in your feelings as a result of such a shift?

1. Please tell me if there is anything that you are glad to have the help of people around you at present.

･What kind of relationship with the person?

･How often and in what situations?

1. How has the opportunity to ask for or receive help from the people around you changed compared to before the COVID-19 pandemic?

･Do you feel any change in your feelings as a result of this change?

1. Please tell me about any other changes in how you interacted with people (i.e. staying at home, online interaction) due to the COVID-19 crisis, which you think were good or bad experiences

**Other Coping Strategies for Mental Health**

･You may have experienced some inconveniences or problems due to the changes in your daily life caused by the COVID-19 pandemic,

1. Since the COCIVD-19 pandemic, have you had opportunities to use help or support from people other than those close to you? (Probe: local government, or NPOs) (If yes)

･How often and in what form did you use the support?

･Did you notice any changes before and after you used the support?

1. What do you feel was the most helpful way for you to cope with the stress during the COVID-19 pandemic?
2. Is there any support that you would like to receive or that you feel would be helpful?

**Snowball Sampling**

･We would like to hear from many people like we did today. If you don't mind, could you introduce us to someone you know who is a single parent?

(If yes)･Would it be okay if I told you that you introduced me?

(Ask for the name and e-mail address of the person introduced)

**Wrap-Up**

This is the end of the interview. Thank you very much for taking time out of your busy schedule to cooperate with our research today. If you have any questions in the future, please feel free to contact me at any time. Also, would it be okay if we contact you again to confirm the results when the results are finalized, or if we have any additional questions?
